# Pauperism in London.—II

**Published:** 1890-01-18

**Authors:** 


### Pauperism in London.—II.

London is divided into thirty unions—thirty separate bodies which, though engaged in the same work of administering to the needs of the destitute poor, have no necessary connection with each other, no common control or basis of action. The parochial system, which Mr. John Morley is so strenuously anxious to restore to its pristine importance all over the country, exists in this matter almost unimpaired in our midst. What is the result? The greatest inequality in every possible detail of administration. Anyone who, like the present writer, has passed a night in a casual ward will have heard some such remark as the following: "I wouldn't have come here if the ward at —— Union hadn't been full. They treat you well there; let you go out directly you've had your breakfast. Here they make you pay for your lodging in work." The regular casual knows his haunts as the accustomed globe-trotter knows the hotels of all countries, and may be seen at an early hour of the afternoon waiting for admission to a ward where the authorities do not demand the horrid penalty of work for the shelter they give. Others, where two or three hours' labour are exacted, are frequented only by the country tramp who does not know his way about town.

Now it is absolutely certain that the parochial system cannot be dispensed with in Poor Law administration; on the contrary, it ought to be pushed to a far greater extent than it has yet reached; so that some member of the board of guardians should be able to give an opinion, founded on local and, if possible, personal knowledge, as to the worth or unworth of each applicant for relief. The parochial system, as practised at Elberfeld, for example, has our strongest commendation.

But above and beyond this strict and systematic surveillance of the parish, is it not necessary to have a central controlling authority unbiassed by local prejudices, unaffected by local interests, to supervise the action of the local guardians? There are many matters of expenditure which such a central board could regulate, and thereby do away with the accusation of jobbery which is so often cast, truly or falsely, at the guardians of various unions. Looking over the lists of contract prices, we must admit that there is every appearance of truth in such statements. We quote some statistics given by Mr. Acworth, who was very much in earnest in pressing forward the necessity for such a central board. "The candles, at 1½d. per lb., with which St. Olave's illumines its work, may be well adapted for their purpose; but if so, it is surely hard upon Lewisham ratepayers that they should have to pay no less than 1s. per lb. Or again, 102s. 8d. per cwt. may be a fit and proper price for arrowroot in St. George's-in-the-East, but in that case the Holborn paupers who are required to swallow an article at 18s. 8d. are much to be condoled with. Variations in lard from 93s. to 37s.; in cocoa from 140s. to 21s.; in currants from 42s. to 20s.; in salt from 50s. to 18s. 6d.; in vinegar from 2s. to 5d.; in pearl barley from 23s. to 9s.; and in split peas from 9s. to 3s. 3d. per cwt.—surely these are wider than occur in ordinary domestic life? Is not the inference obvious, even after all possible allowances have been made, that either the one board is wasting money, or else that the other is not supplying a genuine and wholesome article?"

No sane person will pretend that there is such an enormous difference in price in the same articles in London, taking the metropolitan area at its widest. Even if it were so, if candles of the same quality were nearly ten times as dear at Lewisham as in Southwark, that would simply be a reason for the Guardians of Lewisham, the trusted and presumably trustworthy stewards of the ratepayers' money, buying their candles elsewhere than in the parish. If the Lewisham candles are, on the other hand, ten times better than those of St. Olave's, the paupers of the latter have a just ground of complaint. Equality of treatment should in common honesty go with equality of condition, and if it is not granted we shall certainly find the shrewdest of the destitute classes exercising all their wits to obtain a claim on those unions where they can obtain better food, more comfortable quarters, or a larger dole of outdoor relief. It is useless to blame the paupers; they are only looking after their own interests, as we should probably do in their place. Let us blame the system that makes such inequality possible, and such scheming profitable.

As a matter, then, of justice to the poor, on the one hand, it is advisable to have a central authority, who shall see that on the one hand they shall not be compelled to eat food that is unfit for human consumption, and that money contributed for their benefit shall not be turned away to line the pockets of intriguing and dishonest tradesmen, who charge more than a fair price for the wares they supply. This latter point concerns the ratepayers also, who have a right to be sure that the sum in which they are mulcted shall be honestly expended, and for another reason a central board would be a distinct benefit to them. There are doubtless paupers in South Kensington and rich men in Poplar, but, speaking generally, the first is a rich district, and the second a poor one. Where the poor are most numerous the poor rates will be higher; that is inevitable. It follows, then, that the districts least equal to the burden are taxed the highest for the maintenance of the destitute poor. This is surely hard; nay more, it is unjust. For the work done by, say, an aged dock labourer in his working days benefited not merely the district in which he lived and spent his money, but every part of the metropolis which is concerned, directly or indirectly, in the commerce of the Thames. And to say what part is not would be a difficult task. Moreover, many working-men live in a poorer district than that they work in, and are some distance away. Yet when their working days are done the ratepayers of the former district alone are called upon to support them. Surely this is unfair. London is a many-limbed monster of cities, a Briareus with a hundred arms perhaps, but still a single entity, a corporate whole. As such it must be treated. To regard each limb as a separate creature is stupid and unjust, a wrong to the ratepayers and to the paupers alike. The parochial system answered well enough when master, workmen, and apprentices dwelt under the same roof, or within a street's length of each other. It is still sufficient in the country, where squire and hind live within the same boundaries; but in London and other large cities, where a close interdependence of interests exists between districts miles apart, the survival of the rustic parochial system is anomalous.

For yet one other reason, urged by Mr. Acworth, a central board of control is desirable. At present very little interest is taken by the general public in Poor Law matters. Only the most aggravated instances of mismanagement arouse any interest, and the most energetic newspapers are moved to send a reporter to a meeting of guardians only when some such incident as the death of a pauper under the discipline of a workhouse governor affords an excuse for sensational "copy." How different is it with such institutions as the School Board and the County Council. The eye of public opinion—a troublesome, but, in the main, a wholesome organ—is continually upon them. Their deeds and misdeeds are matter of common talk. The publicity given to all their actions in newspaper reports not only exercises a salutary discipline upon their members, but educates those who read to a clearer knowledge of their aims and work. If this is desirable in matters municipal and educational, surely it is equally desirable in the administration of that wide system of charity known as the Poor Law. Public interest in this matter ought to be aroused. Abuses ought to be rectified, uniformity of treatment insisted on, and it is the ratepayers' duty to see to this. If, as seems likely, this institution of a central board of guardians will lead to this end, let all who seek the welfare of the poor strive to obtain it.

